# Analysis of Nitrification Efficiency and Microbial Community in a Membrane Bioreactor Fed with Low COD/N-Ratio Wastewater

**DOI:** 10.1371/journal.pone.0063059

**Published:** 2013-05-07

**Authors:** Jinxing Ma, Zhiwei Wang, Chaowei Zhu, Shumeng Liu, Qiaoying Wang, Zhichao Wu

**Affiliations:** 1 State Key Laboratory of Pollution Control and Resource Reuse, School of Environmental Science and Engineering, Tongji University, Shanghai, P. R. China; 2 Chinese Research Academy of Environmental Sciences, Beijing, P. R. China; Missouri University of Science and Technology, United States of America

## Abstract

In this study, an approach using influent COD/N ratio reduction was employed to improve process performance and nitrification efficiency in a membrane bioreactor (MBR). Besides sludge reduction, membrane fouling alleviation was observed during 330 d operation, which was attributed to the decreased production of soluble microbial products (SMP) and efficient carbon metabolism in the autotrophic nitrifying community. 454 high-throughput 16S rRNA gene pyrosequencing revealed that the diversity of microbial sequences was mainly determined by the feed characteristics, and that microbes could derive energy by switching to a more autotrophic metabolism to resist the environmental stress. The enrichment of nitrifiers in an MBR with a low COD/N-ratio demonstrated that this condition stimulated nitrification, and that the community distribution of ammonia oxidizing bacteria (AOB) and nitrite oxidizing bacteria (NOB) resulted in faster nitrite uptake rates. Further, ammonia oxidation was the rate-limiting step during the full nitrification.

## Introduction

Membrane bioreactor (MBR) technology is a reliable and promising process in wastewater treatment and reclamation owing to its distinctive advantages over conventional activated sludge (CAS) systems. Of particular significance is that the MBR systems avoid cell washout by retaining complete biomass, which favors the growth of autotrophic nitrifying bacteria and consequently increases the nitrification efficiency, as reported previously [1].

The nitrification pathway of ammonium removal in MBRs is a two-step reaction undertaken by ammonia oxidizing bacteria (AOB) and nitrite oxidizing bacteria (NOB): AOB oxidize ammonium to nitrite in the first step and then NOB oxidize nitrite to nitrate in the following step [2], [3]. Nitrifiers (AOB and NOB) are autotrophic bacteria and could derive energy for growth solely from the oxidation of ammonium/nitrite. However, in conventional MBRs, nitrification is not a strictly independent pathway and carbon oxidation is inevitable during this autotrophic process, which results in a bloom of heterotrophs. Even in an anoxic/oxic MBR, a considerable fraction of the organic carbon is still oxidized aerobically due to endogenous respiration of biomass as well as the leakage of organic carbon to aerobic tanks caused by the high recirculation flow [4], [5].

The unexpected heterotrophic metabolism under aerobic condition, on one hand, consumes a large quantity of influent organic carbon and oxygen. Huge amounts of waste activated sludges (WAS) are produced during this process and their microbial products have been verified as the active component causing membrane fouling in MBRs [6]. In addition, heterotrophs compete with nitrifying bacteria for oxygen and space [3], [7] and the accumulation of heterotrophic waste also inhibits the activities of the *Nitrosomonas* and the *Nitrobacter* group [8]. In the presence of organic carbon, nitrifiers are usually outcompeted by heterotrophs, which eventually cause the nitrification efficiency to decrease [9], [10]. Verhagen and Laanbroek [11] found that under such conditions the nitrifying bacteria were strongly reduced above the critical carbon-to-nitrogen ratios and the numbers of *Nitrosomonas europaea* decreased more than those of *Nitrobacter winogradskyi*.

In light of these findings, we explored a novel approach to improve the process performance and nitrification efficiency in an MBR. Since heterotrophs gain their energy primarily from organic carbon, it is possible to control heterotrophic metabolism by cutting down the external organic carbon supply or reducing the influent COD/N ratio [12], [13]. We hypothesized that operation in a low organic loading mode would result in nitrification stimulation, and that the metabolism (e.g. proliferation) of activated sludge would be altered in this mode, which would consequently influence the operation of MBRs (e.g., membrane fouling). To the best of our knowledge, the information of the effect of influent COD/N ratio on MBR performance and microbial community, especially as applied to low strength municipal wastewater treatment, is very limited.

Therefore, the overarching goal of this study was to evaluate the process performance and nitrification efficiency of an MBR fed with low COD/N-ratio municipal wastewater. 454 high-throughput pyrosequencing was then used to analyze the resulting bacterial population by sequencing the bacterial 16S rRNA gene, allowing us to investigate the population dynamics of the nitrifiers and heterotrophs in MBRs fed with different COD/N-ratio wastewater.

## Materials and Methods

### Lab-scale MBR: Configuration and Operating Conditions

**Figure 1:**
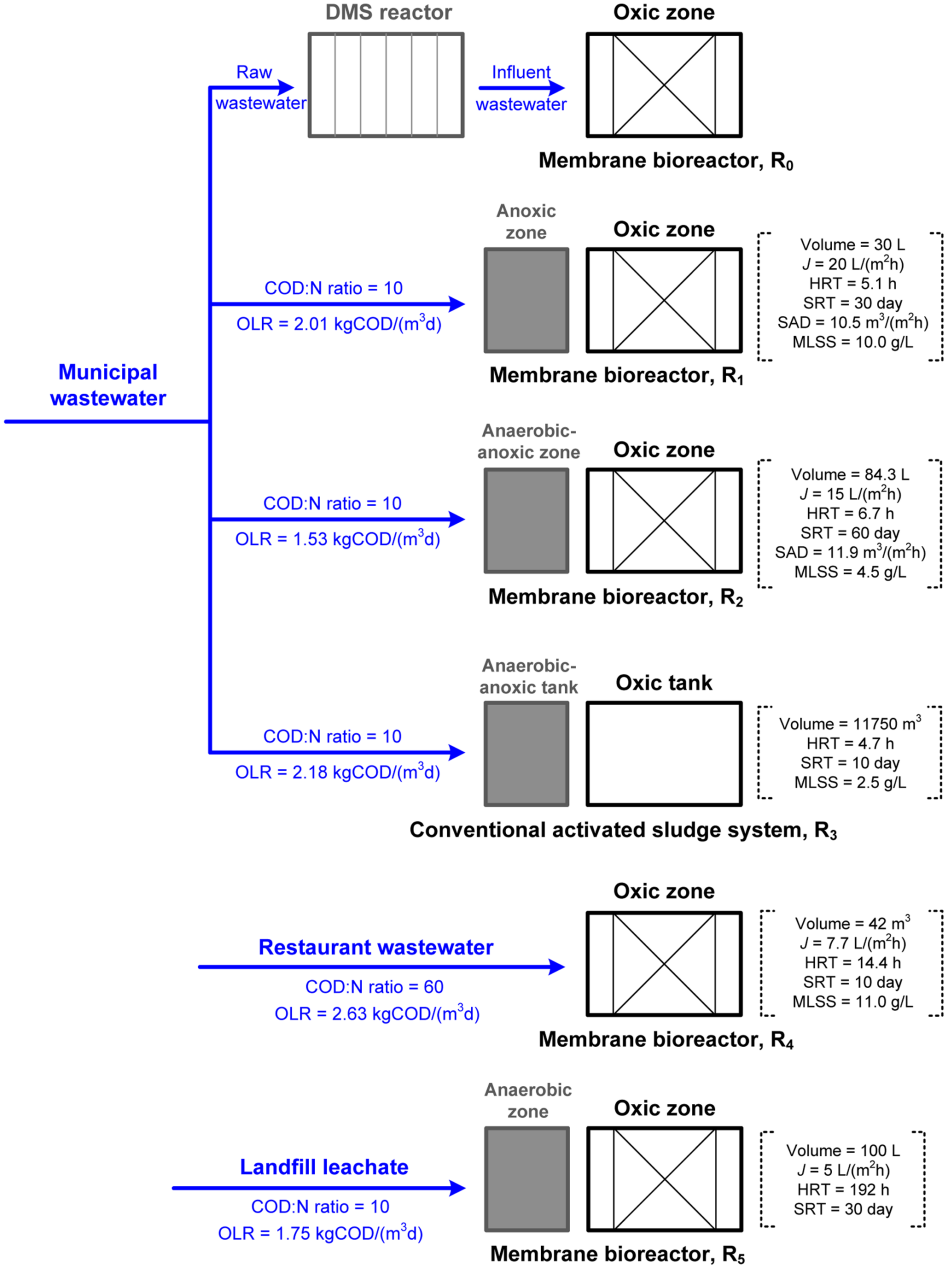
Schematic diagram of R_0_ fed with low COD/N-ratio wastewater and control reactors (R_1_∼R_5_). OLR, *J*, HRT, SRT and SAD represent organic loading rate, permeate flux, hydraulic retention time, and sludge retention time and specific aeration demand per unit projected area of riser zone, respectively.

The lab-scale MBR (R_0_) consisted of a tank with an effective volume of 26 L (Figure S1 in Supporting Information). The influent came from a dynamic membrane separation (DMS) reactor (Figure 1). In our previous work, we have successfully decreased the COD/N ratio of raw wastewater through organic carbon recovery by the DMS reactor [14]. The characteristics of the wastewater are listed in Table 1. Two 40 cm×30 cm flat sheet membrane modules (PVDF, 0.40 µm, Kubota Corporation, Japan) were mounted vertically between two baffle plates located in the tank; the permeate flux (*J*) was set at 18–24 L/(m^2^·h).

**Table 1:** Characteristics of the raw, influent and treated wastewater (Unit: mg/L)^a^.

		Phase I	Phase II	Phase III
Raw wastewater	COD	475.5±175.4	452.0±201.0	337.5±103.5
	TN	44.7±10.5	41.5±11.3	43.5±7.3
Influent wastewater	COD	66.5±23.8	57.3±18.8	75.2±27.8
	TN	20.3±3.2	18.9±2.3	26.7±4.4
	NH_3_-N	17.4±5.2	16.2±3.2	24.5±4.9
Treated wastewater	COD	14.3±14.9	8.8±5.6	17.0±10.0
	TN	19.8±3.5	17.2±5.0	23.6±2.8
	NH_3_-N	n.d.^b^	n.d.	n.d.

a Values are given as mean ± standard deviation and *n* = 18, 11, 13 for Phase I, Phase II and Phase III, respectively.

b n.d.: not detectable.

The operation conditions of R_0_ are summarized in Table 2. Three phases with different hydraulic retention time (HRT) and sludge retention time (SRT) were performed to evaluate the reactor performance and to calculate the activated sludge yield coefficient (*Y*) under a low COD/N ratio. Over the 330 days’ operation, sludge was periodically wasted from the tank to maintain a SRT of 45 d in Phase I, 91 d in Phase II and 182 d in Phase III, respectively. The dissolved oxygen (DO) concentration of the tank was in the range of 1–3 mg/L. Details of the MBR configuration and operation can be found in Supporting Information (Text S1 and Figure S1).

**Table 2:** Operational conditions of R_0_ in three phases.

					COD/N ratio	
Phase	*J*, L/(m^2^·h)	HRT, h	SRT, d	SAD, m^3^/(m^2^·h)	Raw wastewater	Influent wastewater
I	18	4.6	45	10.5	10.8±4.3	3.3±1.0
II	24	3.4	91	14.3	11.3±5.3	3.1±1.0
III	24	3.4	182	14.3	7.8±1.8	3.0±0.6

For a full understanding of the microbial population dynamics resulting from influent COD/N variation, bacterial compositions in five control reactors (R_1_∼R_5_) were also evaluated in this study. The schematic diagram of the reactors can be found in Figure 1. R_1_ and R_2_ ran in parallel with R_0_, which were located in a municipal wastewater treatment plant (R_3_). R_4_ was constructed to treat high COD/N-ratio restaurant wastewater and R_5_ to treat landfill leachate. Membrane bioreactors (R_1_, R_2_, R_4_ and R_5_) were operated in a suction cycle of 10 min followed by 2 min relaxation to alleviate membrane biofouling and a chemical cleaning-in-place procedure (0.5% (v/w) NaClO solution, 2 h duration) was carried out if the trans-membrane pressure reached about 30 kPa during the operation. More information about the control reactors is available in our previous publications [6], [15], [16].

### Calculation Procedures

The filtration resistance of R_0_ over time was calculated using the following equation according to the literature [17]:
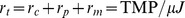
(1)where *r*_t_ is the total filtration resistance (m^−1^), *r*_c_ is the cake layer resistance (m^−1^), *r*_p_ is the pore-clogging resistance (m^−1^), *r*_m_ is the intrinsic membrane resistance (m^−1^), *J* is the permeate flux (m^3^/(m^2^·s)), and TMP is the applied transmembrane pressure (Pa), and *μ* is the permeate viscosity (Pa·s).

The sludge yield coefficient (*Y*, mgVSS/mgCOD) and decay coefficient (*K*_d_, day^−1^) in R_0_ were estimated from the material balance of substrate and biomass, according to the following equation:
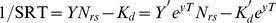
(2)where *N*_rs_ is sludge loading rate (kgCOD/(kgMLVSS·d)), *T* is the temperature (°C), and *Y′* and *y* (or *K*_d_ *′* and *k*) are the Arrhenius constant and exponent of *Y* (or *K*_d_), respectively. *Y* and *K*_d_ values for conventional MBRs were determined according to the MBR book [18] and our previous studies.

Biokinetics of microbial activities in R_0_ inferred from ammonia, nitrite and acetate oxidation were estimated via extant respirometry. Specific oxygen uptake rates (SOUR_A_) and specific ammonia uptake rates (SAUR) related to AOB, SOUR_N_ and specific nitrite uptake rates (SNUR) related to NOB, and SOUR_H_ related to heterotrophs at different temperatures were measured separately using batch assays [19]. Specific nitrification rate (SNR) was determined by the limiting rate of SAUR or SNUR. The detailed measurement and calculation procedures are shown in Supporting Information (Text S1 and Table S1).

### Microbial Diversity Analysis

#### DNA extraction and PCR amplification

Samples for pyrosequencing were obtained from oxic zones of the reactors (R_0_∼R_5_) in August, 2011 and water temperature was about 27°C. R_0_ was operated in Phase I with SRT of 45 d and HRT of 4.6 h. All sludge samples were settled and concentrated onsite and immediately transported to the laboratory for further treatment. DNA extraction was processed using the FastDNA® SPIN Kit for Soil (MP Biomedicals, Solon, OH, USA) according to manufacturer’s protocols. The quantity and quality of the extracted DNA were assessed using a Nano-drop® ND-1000 spectrophotometer (Labtech International, UK). For genetic library construction, DNA from the 5 MBRs (R_0_, R_1_, R_2_, R_4_ and R_5_), and from R_3,_ were each amplified by PCR using primer set 27F (5′-AGAGTTTGATCCTGGCTCAG-3′) and 533R (5′-TTACCGCGGCTGCTGGCAC-3′) for the V1-V3 region of the 16S rRNA gene. The 20 µL PCR mixture contained 4 µL of 5×FastPfu Buffer, 2 µL of 2.5 mM dNTPs, 0.4 µL of each primer (5 µM), 0.5 µL of DNA and 0.4 µL FastPfu Polymerase. The thermocycling steps were as follows: 95°C for 2 min, followed by 25 cycles at 95°C for 30 sec, 55°C for 30 sec, 72°C for 30 sec and a final extension step at 72°C for 5 min. The fused forward primer includes a 10-nucleotide barcode inserted between the Life Sciences primer A and the 27F primer. The barcodes allowed sample multiplexing during pyrosequencing in a single 454 GS-FLX run.

#### 454 high-throughput 16S rRNA gene pyrosequencing

After purification using the UNIQ-10 PCR Purification Kit (Sangon, Shanghai, China) and quantification using a TBS-380 (Turner BioSystems, Inc., USA), a mixture of amplicons was used for pyrosequencing on a Roche massively parallel 454 GS-FLX Titanium sequencer (Roche 454 Life Sciences, Branford, CT, USA) according to standard protocols [20]. To minimize the effects of random sequencing error, low-quality sequences were removed by eliminating those without an exact match to the forward primer, those without a recognizable reverse primer, those with length shorter than 150 bp, and those containing any ambiguous base calls (Ns) [21]. Barcodes and primers were then trimmed from the resulting sequences. Pyrosequencing produced 7818 (R_0_), 6629 (R_1_), 7429 (R_2_), 8265 (R_3_), 9854 (R_4_) and 7944 (R_5_) high-quality V1-V3 tags of the 16S rRNA-gene with an average length of 419 bp.

#### Biodiversity analysis and phylogenetic classification

Initially, sequences were analyzed by performing a BLAST search via the silva106 database at a uniform length of 150 bp and then clustered into operational taxonomic units (OTUs) by setting a 0.03 or 0.05 distance limit (equivalent to 97% or 95% similarity) using the MOTHUR program (http://www.mothur.org/wiki/Main_Page). From the cluster file, the rarefaction curves at *α* of 0.03, 0.05 and 0.10 were generated in MOTHUR for each sample. Taxonomic classification down to the phylum, class, order, and family and genus level was performed using MOTHUR via the silva106 database at a uniform sequence length of 400 bp with a set confidence threshold of 80%. Hierarchical cluster analysis was performed using the gplots package of R (http://www.r-project.org/) in Linux. The Chao linkage method was employed for distance calculation and the complete linkage method for cluster analysis in both coltree and rowtree of heatmap. MEGAN 4.0 software (http://ab.inf.uni-tuebingen.de/software/megan/) was then used to interactively explore the dataset. Each node is labeled by a taxon and the number of reads assigned to the taxon, and the size of a node (the pie chart) is scaled logarithmically to represent the number of assigned reads.

### Analytical Measurements

Analytical measurements of chemical oxygen demand (COD), ammonium (NH_3_-N) and total nitrogen (TN) in raw, influent and treated wastewater, total mixed liquor suspended solids (MLSS), and mixed liquor volatile suspended solids (MLVSS) in the system were performed according to the *Standard Methods* [22]. Protein was measured by the modified Lowry method using bovine serum albumin (BSA) protein as a standard [23]. Carbohydrate was measured according to the phenol-sulfuric acid method with glucose as a standard [24]. TOC and UV_254_ of the filtrate of mixed liquor was quantified by a TOC analyzer (TOC-VCPN, Shimadzu, Japan) and 2802 UV/VIS spectrophotometer (Unico Inc., USA), respectively. Dissolved oxygen (DO) and temperature were monitored by using a DO meter HQ30d with probe LDO10103 (Hach Co., USA) online. Moreover, microscopic examination of the mixed liquor sample was performed according to the protocols [15] twice a week. Aquatic worms’ bloom was defined as the sharp decrease of biomass concentration with the presence of >1000 metazoans per L mixed liquor.

## Results and Discussion

### Process Performance

**Figure 2:**
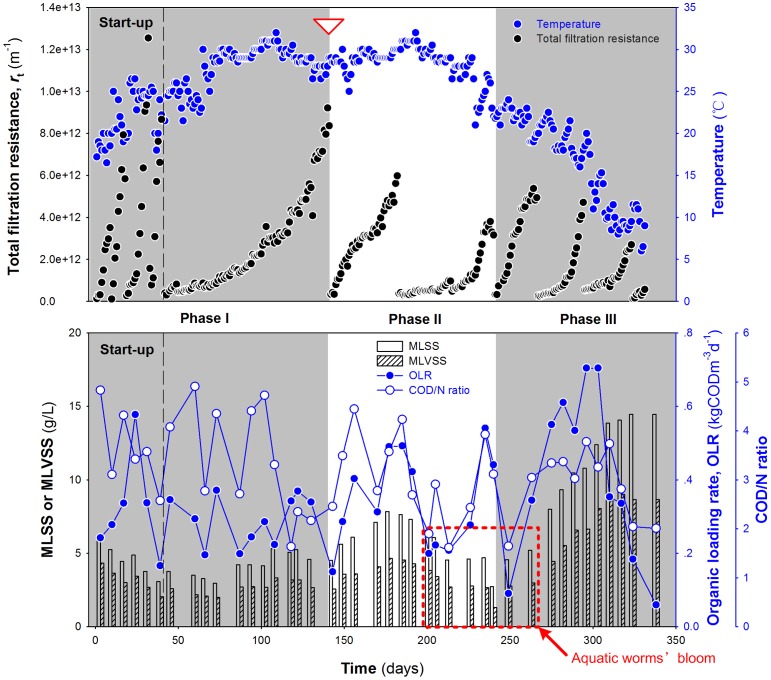
Variations in *r*_t_, biomass concentration, OLR and COD/N ratio during R_0_ operation. (The inverted open triangle indicates the time point of sludge sampling).

At the beginning of the experimental runs, a period of time intended for biomass acclimation (designated as start-up phase in Phase I) was imposed on R_0_. The stable biomass concentration of 4.66±0.48 g MLSS/L, 7.09±0.67 g MLSS/L and 14.60±0.59 g MLSS/L was achieved in Phase I, Phase II and Phase III, respectively (Figure 2). Since a large fraction of influent organic carbon was recovered in the upstream process (Table 1), WAS production involved in the treatment of per ton wastewater was decreased by 60–80%. Moreover, with the feeding strategy adopted, in which a low organic loading rate (OLR) was applied to favor the growth of autotrophic microorganisms, it was inferred that a low COD/N ratio would cause a limiting supply of nutrients for microorganism growth, and could result in a low sludge yield. Relevant literature has documented that an autotrophic community could derive energy for growth from the oxidation of ammonium/nitrite [25], resulting in a thinner microcolony structure in bioreactors [7], [13]. In view of this, we calculated the sludge yield coefficient (*Y*) and decay coefficient (*K*_d_) to evaluate the carbon metabolism in R_0_. *Y* and *K*_d_ refer to microorganism growth and endogenous respiration. OriginPro 8 (OriginLab Corporation, USA) was applied to process the nonlinear curve fit and the results are shown in Figure S2.

In this study, the kinetic parameters were calculated as *Y* = 0.362e^0.001*T*^ mg VSS/mg COD and *K*_d_ = 0.023e^0.006*T*^ day^−1^ using the operational data, and these constants at 20°C were lower than those of conventional MBRs (0.56–0.40 mg VSS/mg COD and 0.08–0.07 day^−1^). This result indicated a limited rate of microorganism growth and biomass decay in a relatively autotrophic run [26]. Under such an oligotrophic environment, organic carbon was likely to be taken up by the starved community to derive energy for system sustainability rather than assimilated for microbial growth, and the significantly decreased *K*_d_ is likely due to the lower aeration intensity [27], [28]. In summary, we preliminarily concluded that the sludge reduction in R_0_ was a result of both source reduction (influent organic carbon) and process reduction (bio-metabolism pathways).

The variations of the total filtration resistance with operation time in the three phases of R_0_ are also shown in Figure 2. After a successful acclimation in the start-up phase, the reactor operation was gradually stabilized with a relatively low rate of increase of *r*_t_. The operation cycles in Phases I and II of R_0_ lasted for about 100 and 60 days, respectively, relatively longer than that in our previous study [15], [16]. Even if the low liquor temperature (5–15°C) caused membrane filtration to deteriorate by the Phase III, the chemical cleaning cycle was still about 30 day. To clarify the mechanism of alleviation of membrane fouling in R_0_, the average content of TOC, protein, carbohydrate and UV_254_, representing dissolved organic matter (DOM) in the supernatant of the mixed liquor, was quantified as 9.40±2.91 mg/L, 11.49±1.11 mg/L, 10.11±3.09 mg/L and 0.090±0.003, respectively (*n* = 7). The DOM level was relatively lower than that of conventional MBRs [6], [15], [16], which presumably mitigated cake layer (or gel layer) formation and pore clogging during membrane filtration. To explain our result, we hypothesize a combined metabolic synergy, i.e., in addition to the decreased production of soluble microbial products (SMP) mentioned above, it is likely that there was an efficient food web (carbon metabolism) in the autotrophic nitrifying community, which ensured maximum heterotrophic utilization of SMP produced by nitrifiers and prevented significant accumulation of nitrifier waste materials, as reported by Kindaichi et al. [9]. In future, further attempts will be made to clarify the principle of SMP production and degradation during autotrophic nitrification in an MBR, and its impact on fouling and membrane filtration.

### Biokinetics of Nitrifiers and Heterotrophs

Table 1 summarizes the average characteristics of the influent and treated wastewater in R_0_. Ammonium could not be detected in the treated water during the experimental operation. In three phases, ammonium was predominately oxidized to nitrate (99.8±0.1%, *n* = 42) rather than nitrite (0.2±0.1%, *n* = 42). The negligible nitrite accumulation (<0.04 mg NO_2_^−^N/(gVSS·h)) during the full-scale nitrification decreased the risk of NO_2_^−^ reduction or its chemical decomposition, which subsequently reduced the transient and stabilized N_2_O and NO emissions, as documented previously [2], [19].

SOUR_H_, SOUR_A_ and SOUR_N_ of the biomass in R_0_ were calculated as 3.91, 1.71 and 4.64 mgO_2_/(gVSS·h) (R^2^ = 0.9985, 0.9994 and 0.9963) at 19.8°C, and 8.47, 2.96 and 8.89 mgO_2_/(g VSS·h) (R^2^ = 0.9922, 0.9960 and 0.9896) at 23.7°C, respectively. The biokinetics of nitrite to nitrate oxidation were significantly (*α* = 0.05) higher than the biokinetics of ammonium to nitrite oxidation, illustrating that ammonia oxidation limited the full-nitrification. SNR was then determined as SAUR. It has been reported that with the SRT prolonged, biomass renovation became slower and enzymatic activity (e.g., SNR) decreased due to competition from the biomass derived from the limiting supply of nutrients [28], while the SNR in R_0_ was calculated as 18.9e^0.059T^ mgN/(gVSS·d) (R^2^ = 0.8370), even higher than that of the conventional MBRs with shorter SRTs [26], [29]. Thus it is possible that the decrease in the influent COD/N ratio inhibited the metabolism of heterotrophs, thereby favoring the nitrifiers in competing for oxygen and scarce substrate (SOUR_A_+ SOUR_N_>SOUR_H_) [11], especially under prolonged SRT conditions.

Further investigation using 454 high-throughput 16S rRNA gene pyrosequencing was performed to compare the microbial diversity and composition (heterotrophs and nitrifiers) of bioreactors fed with different COD/N-ratio wastewater.

### Taxonomic Complexity of the Bacterial Community

Six 16S rRNA gene libraries were constructed from pyrosequencing of R_0_, R_1_, R_2_, R_3_, R_4_ and R_5_ communities with 7818, 6629, 7429, 8265, 9854 and 7944 high-quality reads (average length of 419 bp). The number of sequences was comparable to our previous study [20]. The MOTHUR program was first used to assign these sequence tags into different phylogenetic bacterial taxa and we obtained 1230 (R_0_), 1335 (R_1_), 1668 (R_2_), 1534 (R_3_), 1173 (R_4_) and 781 (R_5_) OTUs at a 3% distance. From the cluster file, the rarefaction curves at *α* of 0.03, 0.05 and 0.10 were generated in MOTHUR for each sample (Figure S3). By comparing the curvature of rarefaction curves, the increase of COD/N ratio from 3.0 to 10.0 (R_0_ *vs* R_1_, R_2_ and R_3_) resulted in the proliferation of the overall bacterial communities, likely due to heterotroph enrichment. The difference in community structures did not actually influence the bioreactor performance with regard to contaminant removal (Table 1), probably due to species functional redundancy [2], [28], i.e functional redundancy appears to be a mechanism for increasing community robustness or responding to changing environments. With the COD/N ratio further increased from 10.0 to 60.5 (R_1_, R_2_ and R_3_ *vs* R_4_), the diversity of the overall bacterial communities was decreased, as observed elsewhere [11], due to the washout of autotrophs. The microbial diversity of biomass in R_5_ was significantly lower, likely in part due to the toxic and high-salty character of its feedwater.

### Comparative Analysis of Bacterial Communities

**Figure 3:**
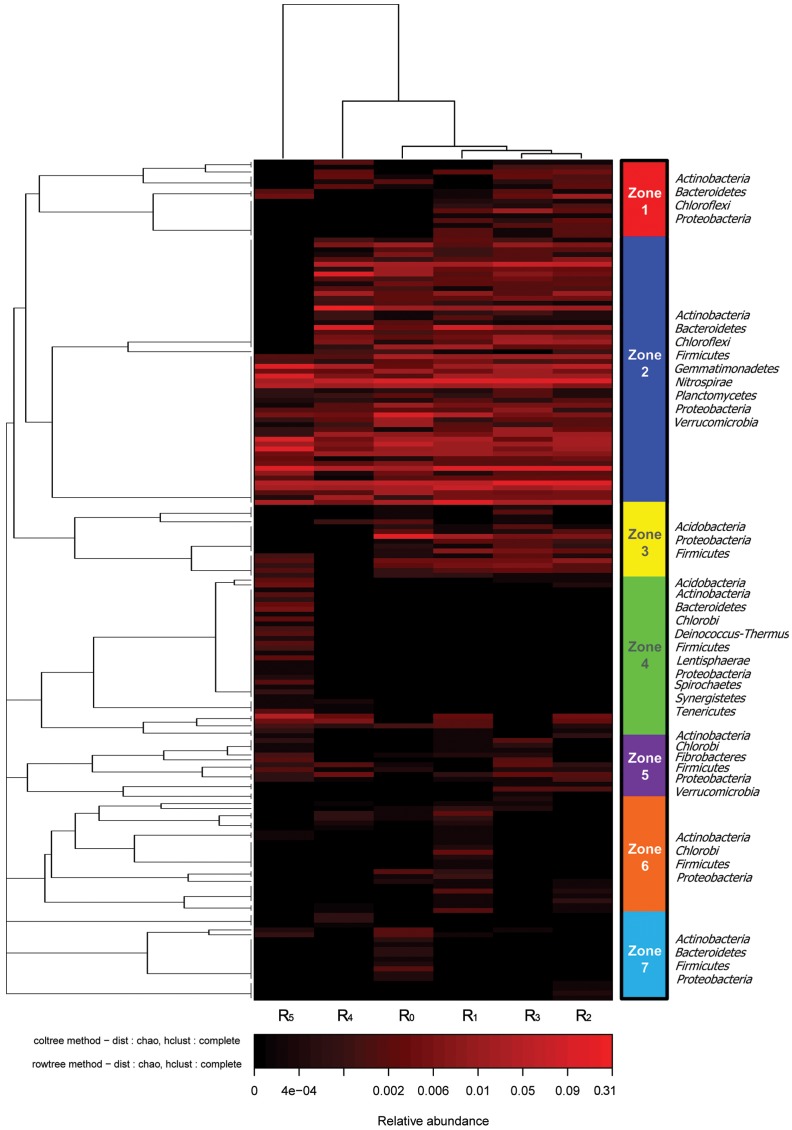
Hierarchical cluster analysis of R_0_–R_5_ bacterial communities. The *y*-axis is the clustering of the 174 most abundant OTUs (3% distance) in reads. The OTUs were divided into seven zones (Z_1_–Z_7_). Sample communities were clustered based on complete linkage method. The color intensity of scale indicates relative abundance of each OTU read. Relative abundance was defined as the number of sequences affiliated with that OTU divided by the total number of sequences per sample.

In order to further compare the population dynamics of heterotrophs, AOB, and NOB with COD/N ratio variations, hierarchical cluster analysis and bacterial taxonomic identification were conducted to illustrate the differences of the six bacterial community structures. In this study, the 174 most abundant OTUs were assigned into seven zones (Z_1_–Z_7_) according to the phylogenetic relationship, and taxonomic complexity of the zones is shown in Figure 3. It could be observed that three clusters were identified from the six bacterial communities by hierarchical cluster analysis: Cluster I (R_0_, R_1_, R_2_ and R_3_), Cluster II (R_4_) and Cluster III (R_5_). The microbial community structures in Cluster I (R_0,_ R_1_, R_2_ and R_3_) exhibited high homology, especially in Z_2_–Z_4_ (Figure 3), and R_5_ was separated from Cluster I and R_4_, having only 0.04% similarity. These results show that sequence homology was mainly determined by feed characteristics (e.g. COD/N ratio and organic substrate composition), and particular bacteria were selectively enriched in their individual bioreactors.

**Figure 4:**
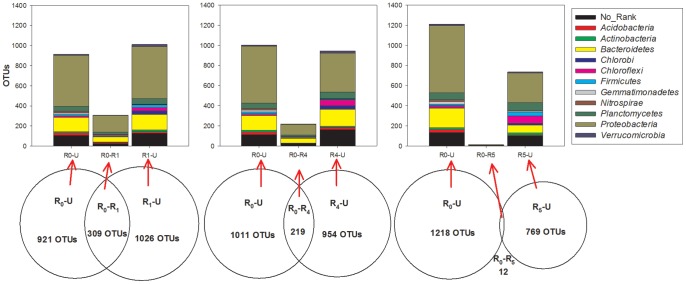
Venn of the bacterial communities of R_0_ *vs* R_1_, R_0_ *vs* R_4_ and R_0_ *vs* R_5_ based on OTU (3% distance), and the taxonomic identities of the shared and unique OTUs at the phylum level (Phyla percentages below 1.0% are not shown). R_0_–U, R_1_–U, R_4_–U and R_5_–U represent the unique R_0_, R_1_, R_4_ and R_5_ communities, and R_0_–R_1_, R_0_–R_4_ and R_0_–R_5_ refer to shared communities.

In contrast to the typical heterotrophic environment, a shift of an MBR from a copiotrophic (e.g. R_1_) to an oligotrophic environment (R_0_) did not increase the diversity of the community, although nitrification stimulation was observed [21]. However, the microbes present in an MBR could derive energy by switching to a more autotrophic metabolism to resist the environmental stress [25]. The total observed OTUs in the R_0_ and R_1_ communities was 2256, with 309 OTUs, or 13.7% of the total, shared by them (Figure 4). Of the 18 identified phyla, *Proteobacteria*, *Bacteroidetes* and No_Rank bacteria accounted for the majority of the unique community composition in R_0_ (55.0%, 15.2% and 11.7%) and R_1_ (50.4%, 14.9% and 12.6%), respectively. Notably, although the organic matter in the influent of R_0_ was lower, the heterotrophic bacteria were still dominant over the autotrophic bacteria, as documented previously [31]. Despite the fact that the population composition of R_0_–U, R_0_–R_1_ and R_1_–U were not significantly different at the 0.05 level (*p* = 0.99985), a marked decrease in the phototropic bacteria in R_0_ was observed compared with R_1_, suggesting more favorable conditions for the growth of the nitrifiers (Figure 4). By contrast, the shared OTUs ratio with R_0_ was decreased by 27.0% with the influent COD/N-ratio increased from 10.0±4.2 (R_1_) to 60.5±13.9 (R_4_). The clearest difference between the unique communities of R_0_ and R_4_ was the different distribution of phyla *Proteobacteria*, *Chlorobi*, *Chloroflexi* and *Nitrospirae* in Z_2_, Z_3_ and Z_5_ in Figure 3. Autotrophic bacteria in phyla *Proteobacteria* and *Nitrospirae* may be depleted in R_4_ under such a copiotrophic environment. Although the influent COD/N ratio was also about 10 in R_5_, its community structure was quite different from the other MBRs’ (Figure 3). Racz has reported that not only the quantity but also the source of the organic carbon affected the make-up of the heterotroph community as well as AOB in mixed cultures [3]. The phyla *Chloroflexi* (9.5%), *Firmicutes* (4.4%) and *Planctomycetes* (10.5%) referring to polysaccharide degradation [32], anaerobic fermentation [21] and sulfated polymeric carbon utilization in the marine environment [33] were enriched in the R_5_ community (Figure 4), suggesting a versatile bio-metabolism of the inert component in the leachate.

**Figure 5:**
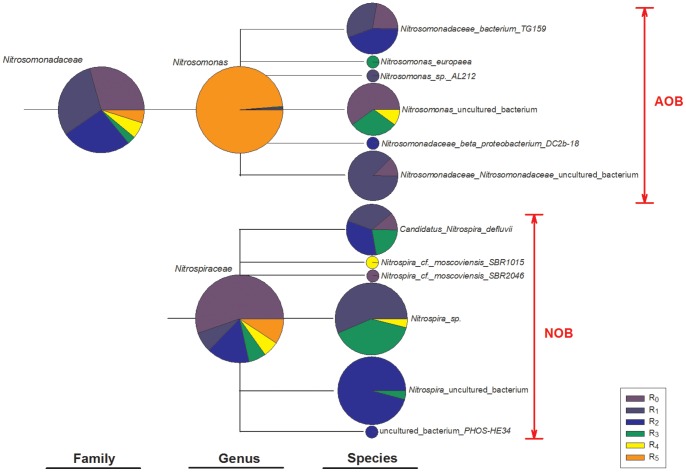
AOB and NOB sequences from R_0_–R_5_ assigned to NCBI taxonomies using BLAST and MEGAN.

Specific comparison of nitrifier communities was conducted using the MEGAN 4.0 software (Figure 5). The ratio of spectrum color in each pie represents the ratio of the relative abundance of reads assigned to the corresponding family, genus or species in R_0_–R_5_. The reads present in the node of one taxon are not assigned to its descendants any more, i.e., these reads are unclassified to the down nodes in the taxonomy. As shown in Figure 5, the dominant AOB species was *Nitrosomonas* and the dominant NOB species was *Nitrospira*, belonging to the orders *Nitrosomonadales* and *Nitrospirales*, respectively. These results are consistent with previous literature characterizing activated sludge systems [3], [31]. From an ecological perspective, then, AOB with a lower half-saturation coefficient for ammonia can thus be enriched in environments such as the full nitrification reactor described here, and the lack of detection of *Nitrobacter* spp.-related NOB was likely due to the propensity of *Nitrospira* spp. to preferentially grow at lower nitrite concentrations [2], [30].

In general, the AOB and NOB were more abundant in MBRs than those in the CAS (R_3_) system (*p*<0.01), which suggests that the complete biomass retention of microfiltration favored the nitrifiers with low growth rate and poor growth yield. With the influent COD/N ratio decreased from 10.0 (R_1_ and R_2_) to 3.0 (R_0_), the population of AOB and NOB was increased by 4.7% and 189.3%. The community distribution of AOB (155 OTUs) and NOB (353 OTUs) in R_0_ resulted in faster nitrite uptake rates and a rate-limiting step of ammonia oxidation during the nitrification. Under a high organic substrate concentration (R_4_), the nitrifiers were outcompeted by the heterotrophs and thus only 31 OTUs of AOB and 41 OTUs of NOB were detected at a 3% distance, which indicated a deterioration of nitrification. In R_5_, the influent organic carbon contained a large fraction of complex protein-like substances (data not shown), and *Nitrosomonas* referring to AOB were highly enriched while NOB species (*Nitrospira*) decreased. This result is consistent with a previous report that the protein-like organic substrate facilitates the growth of AOB [3].

### Conclusions

Sludge reduction and membrane fouling alleviation were induced by the decrease of influent COD/N-ratio. The reduced SMP production and efficient carbon metabolism in the autotrophic nitrifying community facilitated membrane fouling mitigation. The diversity of microbial sequences was mainly determined by feed characteristics, and with a lower COD/N ratio, microbes could derive energy by switching to a more autotrophic metabolism to resist the environmental stress. The enrichment of nitrifiers in the low COD/N-ratio MBR stimulated nitrification, and the community distribution of AOB and NOB resulted in faster nitrite uptake rates and a limiting-rate step of ammonia oxidation during the full-scale nitrification. The results obtained from this study may indicate that decreasing influent COD/N ratio of MBRs (e.g. recovering organic matter from the influent) should be a promising means to improve nitrification efficiency and to alleviate membrane fouling.

## Supporting Information

Figure S1 **Schematic of R_0_ fed with low COD/N-ratio municipal wastewater.**
(TIF)

Figure S2 **Nonlinear curve fit of** ***Y*** **and** ***K*** **_d_, and analysis of variance (ANOVA).**
(TIF)

Figure S3 **Rarefaction curves of OTUs defined by 3%, 5% and 10% distances in R_0_–R_5_ sludge samples.**
(TIF)

Table S1 **Substrate composition for SOURs.**
(DOCX)

Text S1 **More information on MBR configuration/operation and SOUR/SAUR/SNUR determination.**
(DOCX)
