# Supplementary figures and images for: Analysis of Nitrification Efficiency and Microbial Community in a Membrane Bioreactor Fed with Low COD/N-Ratio Wastewater
Source: PLoS One. 2013 May 7;8(5):e63059. doi: 10.1371/journal.pone.0063059 (PMC3646889; doi:10.1371/journal.pone.0063059)

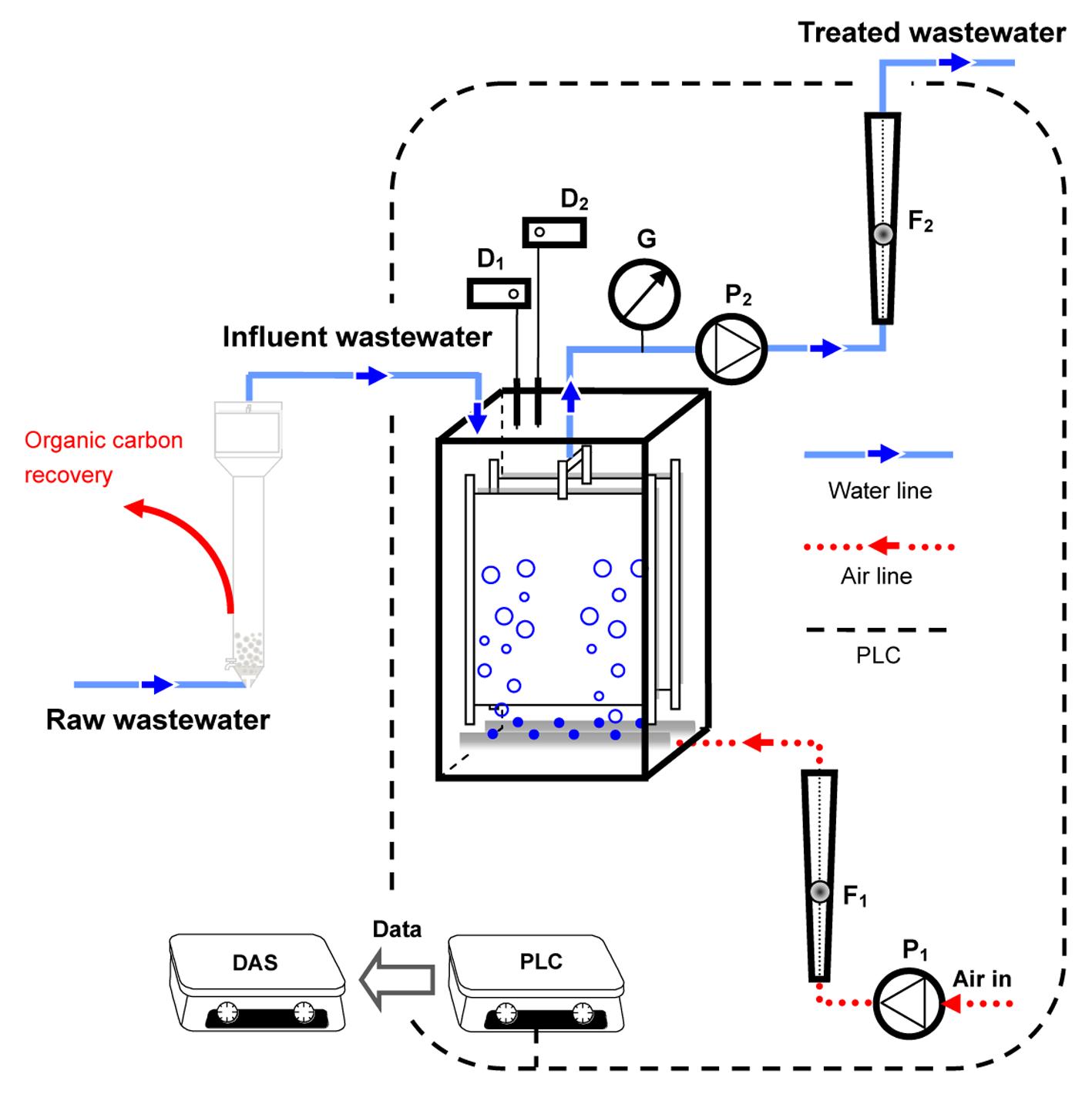

Supplement: Figure S1 — Schematic of R0 fed with low COD/N-ratio municipal wastewater. (TIF) [file pone.0063059.s001.tif]

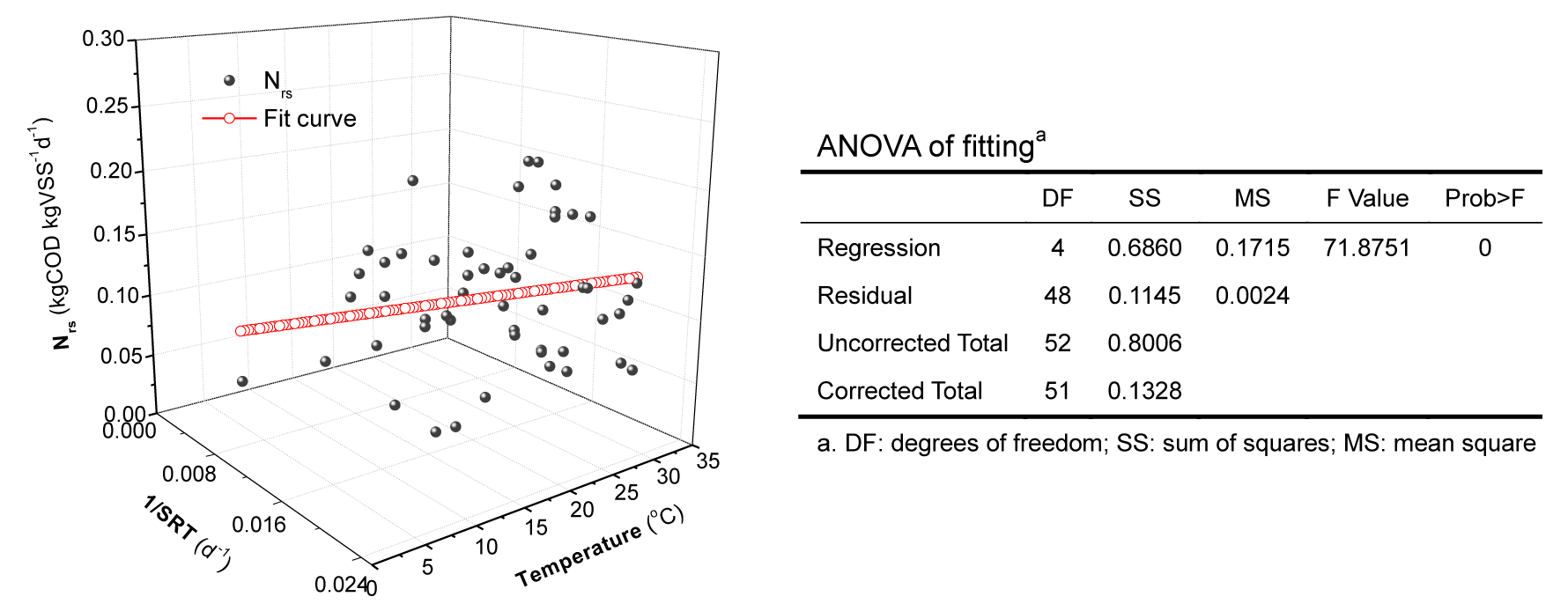

Supplement: Figure S2 — Nonlinear curve fit of Y and K d, and analysis of variance (ANOVA). (TIF) [file pone.0063059.s002.tif]

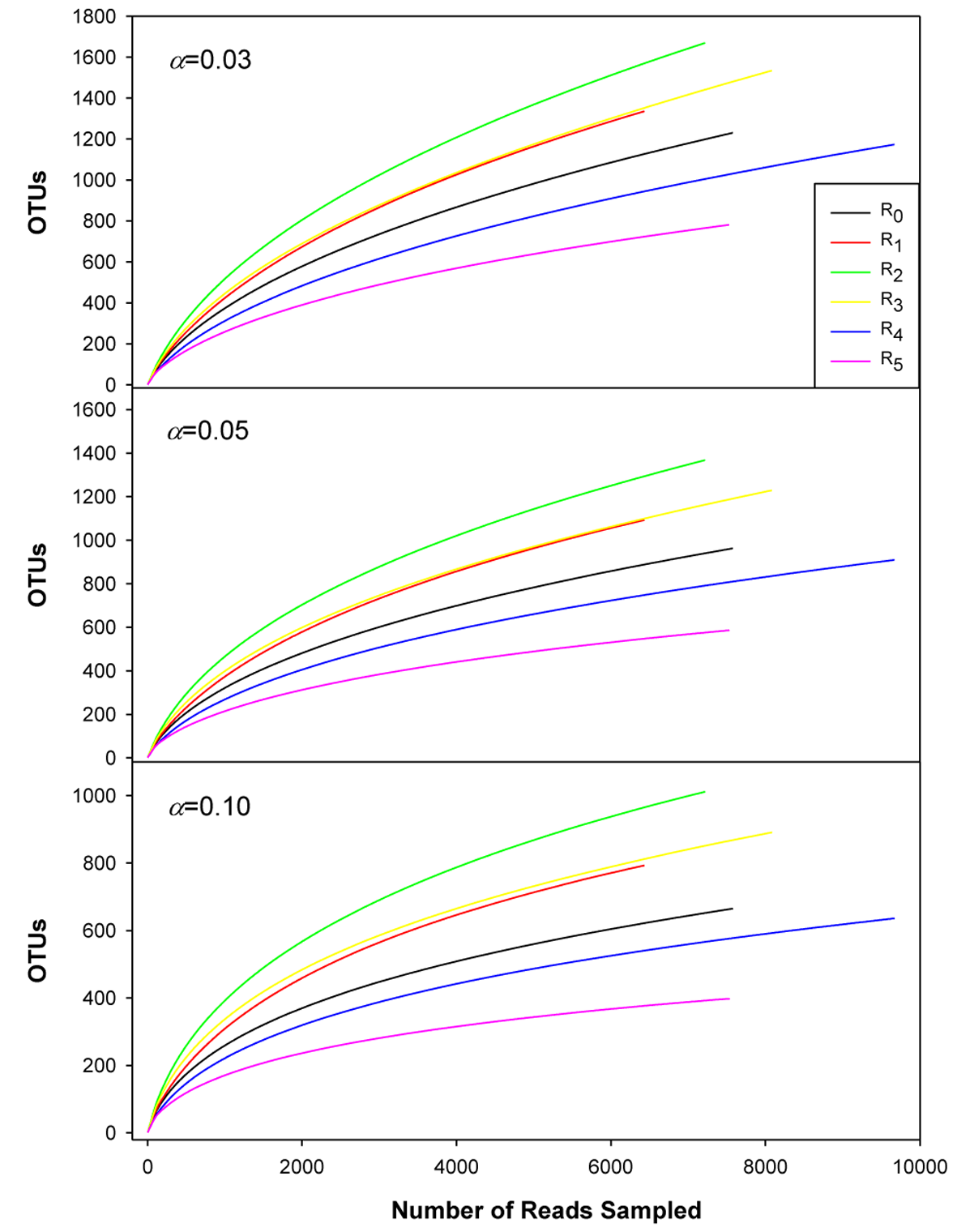

Supplement: Figure S3 — Rarefaction curves of OTUs defined by 3%, 5% and 10% distances in R0–R5 sludge samples. (TIF) [file pone.0063059.s003.tif]
