# Supplementary material for: Analysis of Nitrification Efficiency and Microbial Community in a Membrane Bioreactor Fed with Low COD/N-Ratio Wastewater
Source: PLoS One. 2013 May 7;8(5):e63059. doi: 10.1371/journal.pone.0063059 (PMC3646889; doi:10.1371/journal.pone.0063059)
Supplement: Table S1 — Substrate composition for SOURs. (DOCX) [file pone.0063059.s004.docx]

**Table S1 Substrate composition for SOURs.**

| Items^a^ | SOUR_H_ | SOUR_N_ | SOUR_A_+SOUR_N_^b^ |
| --- | --- | --- | --- |
| NH_3_-N mg/L | -- | -- | 20 |
| NO_2_^-^-N mg/L | -- | 20 | 20 |
| NaHCO_3_ mg/L | 100 | 100 | 100 |
| COD mg/L | 100 | -- | -- |

^a^ NH_4_Cl was added as the source of NH_3_-N, NaNO_2_ as NO_2_-N and sodium acetate as COD. Fe^3+^, Ca^2+^, Mg^2+^ and trace mineral metals were added according to the Standard Method. 1 M phosphate-buffered saline (PBS, pH = 7.0) was employed to adjust the liquor conductivity.

^b^ SOUR_A_ = (SOUR_A_ + SOUR_N_) – SOUR_N_
