# Supplementary material for: Analysis of Nitrification Efficiency and Microbial Community in a Membrane Bioreactor Fed with Low COD/N-Ratio Wastewater
Source: PLoS One. 2013 May 7;8(5):e63059. doi: 10.1371/journal.pone.0063059 (PMC3646889; doi:10.1371/journal.pone.0063059)
Supplement: Text S1 — More information on MBR configuration/operation and SOUR/SAUR/SNUR determination. (DOCX) [file pone.0063059.s005.docx]

**Analysis of Nitrification Efficiency and Microbial Community in a Membrane Bioreactor Fed with Low COD/N-ratio Wastewater**

Jinxing Ma^1^, Zhiwei Wang^1,^*, Chaowei Zhu^2^, Shumeng Liu^1^, Qiaoying Wang^1^, Zhichao Wu^1^

^1^State Key Laboratory of Pollution Control and Resource Reuse, School of Environmental Science and Engineering, Tongji University, Shanghai 200092, PR China

^2^Chinese Research Academy of Environmental Sciences, Beijing 100012, PR China

**Supporting information Text S1**

**1. More information for the MBR (R_0_) configuration and operation**

The lab-scale MBR (R_0_) was located at the Quyang Municipal Wastewater Treatment Plant (WWTP) of Shanghai (Fig. S1). Aeration was monitored by a flow-rate meter and provided by an air diffuser, placed below the membrane modules in the bottom of the reactor. Besides providing oxygen transfer into the liquid phase, the coarse air bubble distribution induced a cross-flow velocity (CFV) along membrane surfaces and avoided stagnant areas in the reactor. The influent pump was controlled by a water level detector to maintain a constant water level in the bioreactor over the course of the experiment. The membrane-filtered effluent was then obtained by suction using a peristaltic pump connected to the modules. The effluent flow rate and TMP were monitored using a water meter and a pressure gauge, respectively. The actuation of pumps and meters in the system was controlled through a programmable logic controller (PLC) connected to a computer for data acquisition.

A start-up phase in Phase I for the acclimation of activated sludge was introduced at the beginning of the experimental runs. This reactor was inoculated with suspended biomass from a pilot-scale MBR that had been operated for several years. Over the course of the experimental operation, a suction cycle of 10 min followed by 2 min relaxation (no suction) was employed and a chemical cleaning-in-place procedure (0.5 % (v/w) NaClO solution, 2 h duration) was carried out if the TMP reached about 30 kPa during the operation. The MBR was operated under ambient conditions (5.0-31.0 °C).

**2. Calculation procedures for SOUR, SAUR and SNUR [1-5]**

**SOUR:** Initially, sludge samples from R_0_ were processed three times as follows: washed with DI water, centrifuged at 3000 rpm for 5 min, and drained through decantation of the supernatant. The processed samples were diluted to 2 mg MLVSS/L and aerated with pure oxygen before being transferred to respirometric bottles, and then tightly capped with no headspace. At a predetermined time, an aliquot of substrate (Table S1) was added using a 10 mL glass syringe. The decrease of DO in the respirometric vessel was measured by a DO meter and continuously monitored at 0.2 Hz by an interfaced personal computer. The oxygen uptake rate was calculated by a linear regression analysis and then quantified to SOUR based on MLVSS. The classification of SOURs was determined by the substrate composition (Table S1).

**SAUR:** The mixture samples processed by the **SOUR** pretreatment procedure were then transferred to a 1L batch reactor. DI water with sufficient amounts of NH_4_Cl and NaHCO_3_ solutions were added into the reactor to a total liquid volume of 1L and to a final concentration of 20 mg NH_3_-N/L and 100 mg NaHCO_3_/L. The mixed liquid of all batches was continuously aerated by air. Sodium azide (NaN_3_), a selective inhibitor of nitrite oxidation, was also added to inhabit the oxidation of NO_2_^-^ to NO_3_^-^. The mixed liquid samples were harvested, filtered, and analyzed immediately at defined intervals, according to the Standard Method. The ammonia uptake rate was calculated by a linear regression analysis and then quantified to SAUR based on MLVSS.

**SNUR:** SNUR was determined using a procedure similar to that used for SAUR. NaNO_2_ at a concentration of 20 mg NO_2_^-^-N/L was used instead of NH_4_Cl, and no NaN_3_ was added to inhibit the nitrite oxidation.

**References:**

1. Butler MD, Wang YY, Cartmell E, Stephenson T (2009) Nitrous oxide emissions for early warning of biological nitrification failure in activated sludge. Water Research 43: 1265-1272.

2. Hu Z, Chandran K, Grasso D, Smets BF (2002) Effect of nickel and cadmium speciation on nitrification inhibition. Environmental Science & Technology 36: 3074-3078.

3. Huang LN, De Wever H, Diels L (2008) Diverse and Distinct Bacterial Communities Induced Biofilm Fouling in Membrane Bioreactors Operated under Different Conditions. Environmental Science & Technology 42: 8360-8366.

4. Liang ZH, Das A, Beerman D, Hu ZQ (2010) Biomass characteristics of two types of submerged membrane bioreactors for nitrogen removal from wastewater. Water Research 44: 3313-3320.

5. Lovley DR, Phillips EJP (1988) Novel mode of microbial energy metabolism: organic carbon oxidation coupled to dissimilatory reduction of iron or manganese. Applied and Environmental Microbiology 54: 1472-1480.
